# *In vitro* and *in vivo* antimicrobial activity of the fungal metabolite toluquinol against phytopathogenic bacteria

**DOI:** 10.3389/fmicb.2023.1221865

**Published:** 2023-07-31

**Authors:** Dawoon Chung, Hoa Thi Nguyen, Nan Hee Yu, Woon-Jong Yu, Yong Min Kwon, Seung Seob Bae, Grace Choi, Jin-Cheol Kim

**Affiliations:** ^1^Department of Microbial Resources, National Marine Biodiversity Institute of Korea, Seocheon, Republic of Korea; ^2^Plant Healthcare Research Institute, JAN153 Biotech Incorporated, Gwangju, Republic of Korea; ^3^Center of Organic Biochemistry, Vietnam Institute of Industrial Chemistry, Ha Noi, Vietnam

**Keywords:** toluquinol, patulin, gentisyl alcohol, antimicrobial activity, phytopathogenic bacteria, *Penicillium griseofulvum*, bacterial wilt of tomato, bacterial leaf blight of rice

## Abstract

**Introduction:**

Bacterial plant diseases cause tremendous economic losses worldwide. However, a few effective and sustainable control methods are currently available. To discover novel and effective management approaches, we screened marine fungi for their antibacterial activity against phytopathogenic bacteria *in vitro* and *in vivo*.

**Methods:**

We screened the culture broth of 55 fungal strains isolated from various marine sources (seawater, algae, and sediment) for their *in vitro* antibacterial activity using the broth microdilution method. Then, only the fungal strain (designated UL-Ce9) with higher antibacterial activity *in vitro* was tested in an *in vivo* experiment against tomato bacterial wilt. The active compounds of UL-Ce9 were extracted using ethyl acetate, purified by a series of chromatography, and the structure was elucidated by nuclear magnetic resonance spectroscopy. Pesticide formulations of toluquinol were prepared as soluble concentrates and wettable powder. The disease control efficacy of toluquinol formulations was evaluated against blight of rice and the bacterial wilt of tomato.

**Results and discussion:**

The culture broth of UL-Ce9 showed high antibacterial activity against *Agrobacterium tumefaciens, Ralstonia solanacearum*, and *Xanthomonas arboricola* pv. *pruni in vitro*, and we selected UL-Ce9 for the *in vivo* test. The UL-Ce9 culture broth completely suppressed the bacterial wilt of tomato at a dilution of 1:5. The phylogenetic analysis identified UL-Ce9 as *Penicillium griseofulvum*, and the antibacterial metabolites were revealed as patulin, gentisyl alcohol, and toluquinol, all of which were associated with the biosynthetic pathway of the mycotoxin patulin. Patulin exhibited the highest antibacterial activity against 16 phytopathogenic bacteria *in vitro*, followed by toluquinol and gentisyl alcohol. As patulin is toxic, we selected toluquinol to investigate its potential use as a pesticide against bacterial plant diseases. Compared with the chemicals currently being applied in agriculture (streptomycin and oxytetracycline), toluquinol formulations exhibited similar and higher control efficacies against bacterial leaf blight of rice and bacterial wilt of tomato, respectively. To the best of our knowledge, this is the first report of the antibacterial activity of toluquinol against phytopathogenic bacteria. Our results suggest that toluquinol is a potential candidate for the development of novel and effective pesticides for the management of bacterial plant diseases.

## 1. Introduction

Phytopathogenicbacteria infect a broad range of plants and cause wilt, spots, tissue rot, blight, cankers, and imbalanced plant growth (Mansfield et al., 2012). Approximately 150 bacterial species are associated with plant diseases, and the representative genera include *Acidovorax, Agrobacterium, Burkholderia, Clavibacter, Erwinia, Pectobacterium, Pseudomonas, Ralstonia, Streptomyces, Xanthomonas*, and *Xylella* (Kannan et al., 2015). For example, *Ralstonia solanacearum*, a causative agent of bacterial wilt of tomato, is one of the most destructive phytopathogenic bacteria worldwide because of its wide geographical distribution and host range (Mansfield et al., 2012; Rivera-Zuluaga et al., 2023). It is a soil-borne pathogen that infects ~200 plant species belonging to more than 50 families. Bacterial diseases in food-producing plants cause a reduction in crop yield and quality and economic losses of at least 1 billion US dollars worldwide annually (Kannan et al., 2015). *Xanthomonas oryzae* pv. *oryzae*, a causative agent of bacterial leaf blight, is responsible for yield losses of 10%−50% (Mansfield et al., 2012). Climate change and environmental pollution exacerbate plant diseases caused by phytopathogenic bacteria (Martins et al., 2018).

A few effective methods are currently available to manage bacterial plant diseases. Copper-based pesticides are generally used to treat plant diseases, but the application of these compounds has become restricted in many countries, such as those of the EU, owing to environmental concerns (Lamichhane et al., 2018). Antibiotics such as streptomycin and oxytetracycline are used to treat bacterial plant diseases. However, antibiotics are relatively expensive, and their long-term and frequent use can lead to the development of antibiotic resistance in plant pathogens (Sundin et al., 2016; Sundin and Wang, 2018). Therefore, the discovery of alternative strategies for controlling phytopathogenic bacteria is imperative (Puigvert et al., 2019).

Marine fungi are relatively unexplored microorganisms for biotechnological applications compared with terrestrial fungi. To adapt to extreme conditions in marine environments such as a wide range of temperatures, hydrostatic pressure, and salinity, marine microorganisms including marine fungi can produce secondary metabolites distinct from their terrestrial counterparts (Xie et al., 2018). Marine fungi are important sources of natural compounds with various bioactivities. Several marine fungi have been reported to produce bioactive compounds with antimicrobial, antiviral, anticancer, anti-inflammatory, and antioxidant activities (Ameen et al., 2021).

Only a few studies have been performed to identify anti-phytopathogenic compounds in marine fungi. For example, stemphyperylenol and alterperylenol, identified from marine *Alternaria* spp., exhibit antifungal and antibacterial activities against phytopathogens such as *Alternaria brassicicola* and *Clavibacter michiganensis*, respectively (Zhao et al., 2018). Pleosporalone B, isolated from marine *Pleosporales* sp. CF09-1, displays antifungal activity against *A. brassicicola* and *Fusarium oxysporum* (Cao et al., 2019). In addition, 3-decalinoyltetramic acid derivatives from marine *Fusarium equiseti* D39 inhibit the growth of the phytopathogenic bacterium *Pseudomonas syringae* (Zhao et al., 2019). However, antibacterial compounds from marine fungi against phytopathogenic bacteria remain largely unknown.

This study aimed to discover antibacterial compounds from marine fungi to control bacterial plant diseases. We have screened marine fungi for antibacterial activity against phytopathogenic bacteria, and the culture broth of the marine algae-derived strain UL-Ce9 showed high activity *in vitro* and *in vivo*. Antibacterial compounds from UL-Ce9 were identified, and pesticide formulations were prepared using one of the compounds. Finally, the disease control efficacy of the formulations against bacterial wilt of tomato and leaf blight of rice was investigated.

## 2. Materials and methods

### 2.1. Sample collection and fungal isolation

Marine algae were collected from the intertidal region of Ulleungdo, Gyeongsangbuk-do, Republic of Korea (37°27′36.24″N, 130°51′42.69″E). Fungi were isolated and cultivated as described previously (Chung et al., 2021a). In brief, marine algal samples were cut into segments and grown in potato dextrose agar (PDA; BD, Franklin Lakes, NJ, USA) supplemented with 3% sea salt, 0.01% (w/v) ampicillin, and 0.01% (w/v) streptomycin. After cultivation at 20°C for 14 days, fungal isolates were obtained, stored in a 20% glycerol solution at −80°C, and deposited in the National Marine Biodiversity Institute of Korea (MABIK).

### 2.2. Preparation of fungal culture broth

The fungal culture broth was prepared as described previously (Chung et al., 2021b). In brief, five agar plugs were obtained from the fungal colonies grown in PDA at 28°C for 5 days using a cork borer and cultured in 100 ml of potato dextrose broth (PDB; BD) at 28°C for 14 days. The cell-free supernatants (fungal culture broth) were collected by passing them through Miracloth (Millipore, Billerica, MA, USA), followed by 0.22-μm pore size syringe filtration (Corning, New York, USA), and stored at −20°C until use.

### 2.3. Preparation of phytopathogenic bacteria

Sixteen phytopathogenic bacteria (*Acidovorax avenae* subsp. *cattleyae, Acidovorax konjaci, Agrobacterium tumefaciens, Burkholderia glumae, C. michiganensis* subsp. *michiganensis, Erwinia amylovora, Erwinia pyrifoliae, Pectobacterium carotovorum* subsp. *carotovorum, Pectobacterium chrysanthemi, P. syringae* pv. *actinidiae, P. syringae* pv. *lachrymans, R. solanacearum, Xanthomonas arboricola* pv. *pruni, Xanthomonas axonopodis* pv. *citri, Xanthomonas euvesicatoria*, and *X. oryzae* pv. *oryzae*) were used for antibacterial activity tests *in vitro* and *in vivo* as described in a previous study (Le et al., 2021). The bacterial names, related plant diseases, and cultivation conditions are listed in Table 1. The bacterial strains were cultured in tryptic soy agar (TSA; BD) and tryptic soy broth (TSB; BD) at 30°C unless indicated otherwise.

**Table 1:** Phytopathogenic bacteria examined in this study.

**Bacterial species**	**Plant diseases (References)**	**Incubation temperature (°C)**	**Incubation periods (day)**
*Acidovorax avenae* subsp. *cattleyae*	Bacterial brown spot of orchid (Khamtham and Akarapisan, 2019)	30	1
*Acidovorax konjaci*	Leaf blight of konjac (Goto, 1983)	30	1
*Agrobacterium tumefaciens*	Crown gall tumor (Mansfield et al., 2012)	30	1
*Burkholderia glumae*	Bacterial panicle blight (Ortega and Rojas, 2021)	30	1
*Clavibacter michiganensis* subsp. *michiganensis*	Bacterial wilt and canker of tomato (Gartemann et al., 2021)	30	1
*Erwinia amylovora*	Fire blight of apple (Mansfield et al., 2012)	30	1
*Erwinia pyrifoliae*	Fire blight of pear (Rhim et al., 1999)	30	1
*Pectobacterium carotovorum* subsp. *carotovorum*	Soft rot of crop plants (Mansfield et al., 2012)	30	1
*Pectobacterium chrysanthemi*	Soft rot (Lin et al., 2015)	30	1
*Pseudomonas syringae* pv. *actinidiae*	Bacterial canker (Scortichini et al., 2012)	25	1
*Pseudomonas syringae* pv. *lachrymans*	Angular leaf spot of cucumber (Harighi, 2007)	25	1
*Ralstonia solanacearum*	Bacterial wilt of tomato (Mansfield et al., 2012)	30	2
*Xanthomonas arboricola* pv. *pruni*	Bacterial leaf spot of fruit (Lopez-Soriano et al., 2016)	30	2
*Xanthomonas axonopodis* pv. *citri*	Bacterial blight of cassava (Mansfield et al., 2012)	30	2
*Xanthomonas euvesicatoria*	Bacterial leaf spot of tomato and pepper (Dhakal et al., 2019)	30	2
*Xanthomonas oryzae* pv. *oryzae*	Bacterial leaf blight of rice (Mansfield et al., 2012)	30	2

### 2.4. Antibacterial activity assay of the culture broth of marine fungi

We isolated 55 marine fungi from various sources (seawater, algae, and sediment) and tested for *in vitro* antibacterial activity using the broth microdilution method as described in a previous study (Nguyen et al., 2018). Three phytopathogenic bacteria, namely, *A. tumefaciens* (At), *R. solanacearum* (Rs), and *X. arboricola* pv. *pruni* (Xap), were used to examine the antibacterial activity of the fungal filtrates. These bacterial strains were incubated in TSB at 25 or 30°C (Table 1) at 150 rpm for 1 or 2 days, and the bacterial cultures were diluted to obtain an optical density of 0.1 (ca. 1 × 10^8^ CFU/ml) at 600 nm.

One hundred eighty and 100 μl of TSB containing an individual phytopathogenic bacterium at 1 × 10^5^ CFU/ml (except for 1 × 10^6^ CFU/ml for *Xanthomonas* spp.) were pipetted into the first and the second well of a 96-well microtiter plate, respectively. Twenty microliters of the fungal culture filtrate was added to the first well to treat the phytopathogenic bacterium with 10% (v/v) fungal broth. Then, 100 μl of the solution in the first well was transferred to the second well by two-fold dilution to make 5% (v/v) fungal broth. One hundred microliters of the solution in the second well was discarded to make a final volume per well of 100 μl. The streptomycin sulfate solution in H_2_O (by serial two-fold dilutions at a range from 0.78 to 100 μg/ml) and 10% PDB were added to TSB containing phytopathogenic bacteria as positive controls for antibacterial activity and microbial growth, respectively. In addition, 10% PDB added to TSB without bacterial inoculation was included as a negative control. After incubation at 30°C for 72 h, bacterial growth was evaluated by the naked eye and a microplate reader (HIDEX; Turku, Finland) at 600 nm. The minimum inhibitory concentration (MIC) was defined as the lowest concentration of the culture broth capable of inhibiting bacterial growth. This experiment was performed in triplicate for each sample.

Only the fungal strain showing higher antibacterial activities *in vitro* was selected and examined for *in vivo* antibacterial activity against tomato bacterial wilt caused by Rs. One h after the inoculation of Rs in tomato plants, 20 ml of the diluted fungal culture broth (dilutions: 1:5, 1:15, and 1:45) was applied to the plants by soil drenching. Soil drenching is a common application method for pesticides to treat bacterial wilt caused by a soil-borne pathogen, Rs (Lee et al., 2012). The preparation of bacterial inoculum and the rest of the experimental procedures are described in Section 2.8.

Patulin biosynthetic compounds (patulin, gentisyl alcohol, toluquinol, *m*-cresol, *m*-hydroxybenzyl alcohol, *m*-hydroxybenzaldehyde, *m*-hydroxybenzoic acid, gentisyl aldehyde, and gentisic acid) were purchased from Sigma-Aldrich Co. (St. Louis, MO, USA). To assess the MIC against the phytopathogenic bacteria *in vitro*, stock solutions of all the compounds were prepared at 20 mg/ml using methanol.

### 2.5. Fungal identification

Based on the results of the *in vitro* antimicrobial activity against phytopathogenic bacteria, strain UL-Ce9 was selected for fungal identification because this strain showed higher activity than other fungal strains tested. The isolation of fungal genomic DNA, polymerase chain reaction (PCR), sequencing of genetic markers, and phylogenetic analysis were performed as described in a previous study (Chung et al., 2021b). UL-Ce9 conidia were cultured in PDB at 28°C, 150 rpm for 3 days. Genomic DNA was extracted from the harvested, frozen, and ground mycelia using phenol:chloroform:isoamyl alcohol (25:24:1) (Sigma-Aldrich). PCR was conducted using the primers Bt2a (5′-GGTAACCAAATCGGTGCTGCTTTC-3′) and Bt2b (5′-ACCCTCAGTGTAGTGACCCTTGGC-3′) to amplify the beta-tubulin gene (*benA*) (Visagie et al., 2014). The 401 bp-PCR products were purified using a gel extraction kit (Qiagen, Hilden, Germany) and sequenced using the same primers, as described in a previous study (Chung et al., 2021b).

For phylogenetic analysis, closely related sequences were identified using the *benA* sequence as a query for a BLAST search in GenBank, aligned, and edited manually using the MEGA 6 software (Tamura et al., 2011). The aligned sequences were subjected to neighbor-joining analysis, followed by bootstrapping with 1,000 replicates in MEGA 6.

### 2.6. Extraction and isolation of active compounds from UL-Ce9

To isolate the active compounds from the fungal strain UL-Ce9, the fungus was incubated in 1 L of PDB at 28°C for 14 days and filtered using four layers of sterile cheesecloth. The culture supernatants were partitioned sequentially twice with ethyl acetate (EA, 1 L) and butanol (1 L). Water in the EA extraction solution was removed by adding Na_2_SO_4._ Two organic solvents and one aqueous layer were concentrated to dryness using a rotary evaporator. The antibacterial activities of the individual extracts were examined against Rs, and the extract with the lowest MIC value (highest antibacterial activity) was selected for the next steps to identify antibacterial metabolites.

The EA extract (2.6 g) was subjected to column chromatography in a silica gel column (4.5 cm diameter and 58 cm length, Kiesel gel 60, 230 g, 70–230 mesh; Merck, Darmstadt, Germany) and eluted with CHCl_3_/methanol [9/1 (v/v), 800 ml]. All collected fractions were subjected to thin-layer chromatography (TLC; Kiesel gel 60 GF 254, 0.25 mm layer thickness, Merck) using CHCl_3_/methanol (9/1, v/v) as a developing solvent system. Fractions (F1–F6) with similar patterns were pooled and assayed *in vitro* for the growth inhibition of Rs.

The fractions showing antibacterial activity were recombined based on similar TLC patterns and used for TLC bioautography (Wang et al., 2021). The fractions were separated in a thin-layer plate (Kisel gel 60 GF 254, 0.25 mm layer thickness, Merck) with the developing solvent CHCl_3_/methanol (9/1, v/v). The TLC plate was sterilized using ultraviolet (UV) light and placed on TSA inoculated with Rs (1.0 × 10^6^ CFU/ml). After 3 days of incubation at 30°C, the antibacterial activity of the fractions was confirmed.

To isolate the antibacterial compounds (CP**1**, CP**2**, and CP**3**), the fractions were subjected to prep-TLC (Kiesel gel 60 GF 254, 0.5 mm thickness, Merck). The developing solvents CHCl_3_/MeOH (9/1, v/v) and CHCl_3_/MeOH (8/2, v/v) were used to isolate CP**1** and CP**2**, respectively. The purity of the isolated compounds was validated by high-performance liquid chromatography (HPLC, Waters Corp., Milford, Massachusetts, United States). Compounds CP**1**–CP**3** were subjected to HPLC on an Atlantis^®^ T3 column (5 μm, 4.6 × 250 mm, Waters Corp.) with 0.1% trifluoroacetic acid (Sigma-Aldrich) in water (eluent A) and 0.1% trifluoroacetic acid in acetonitrile (eluent B) as a mobile phase at a flow rate of 1 ml/min. Detection was performed using a Waters 996 detector set at 254 nm.

To determine the chemical structures of CP**1**–CP**3**, ^1^H-NMR (nuclear magnetic resonance) and ^13^C-NMR spectra were measured in methanol-d_4_ (Cambridge Isotope Laboratories, Inc., Tewksbury, MA, USA) using a Bruker Avance III HD 500 MHz instrument (Bruker BioSpin GmbH, Rheinstetten, Germany).

### 2.7. Preparation of toluquinol formulations

Two types of formulations, namely, a wettable powder (T-WP) and a soluble concentrate (T-SL), were prepared using toluquinol (also known as methylhydroquinone; Sigma-Aldrich). To prepare T-WP, 20 g of toluquinol suspended in a minimal volume of acetone was combined with 15 g of synthetic hydrated silicon dioxide (white carbon; Rhodia Asia Pacific Pte. Ltd., Singapore). After evaporating the acetone in a fume hood, 5 g of CR-100 (a wetting agent; Yoosung Chemical R&T Co. Ltd.), 5 g of CR-SDS (a surfactant; Yoosung Chemical R&T Co. Ltd.), and 55 g of kaolin (a dispersant) were added. Finally, the mixture was ground using an electric powder grinder. To prepare T-SL, 10 g of toluquinol suspended in 40 ml of ethanol was mixed with 20 g of CR-MC33 (an emulsifier; Yoosung Chemical R&T Co. Ltd.) and water to obtain 100 ml of the final product.

### 2.8. Disease control efficacy against bacterial leaf blight of rice

Rice seeds (8 g) were treated with a sterilizer (1:2,000 diluted Sportak EC (Prochloraz 25%), Hankook Samgong, Seoul, Korea) at 30°C for 1 day and soaked in freshwater at 30°C for 4 days. The seeds (5 seeds/pot) were sown in plastic pots (8 cm in diameter and 7 cm in height) and grown under a light regime of 14-h light/10-h dark at 28–36°C and 90% relative humidity for 6 weeks.

The causative agent of bacterial leaf blight of rice *X. oryzae* pv. *oryzae* (Xoo) was cultured in TSA at 30°C for 2 days, and the cells were resuspended in 10 mM MgCl_2_ solution (pH 7.0). Cell density was measured using a UV spectrophotometer at 600 nm, and the bacterial inoculum suspension was adjusted to 2.0 × 10^7^ CFU/ml. One day before the inoculation of Xoo, 20 ml of diluted T-WP (1:1,000, 1:2,000, and 1:4,000 dilutions), T-SL (1:500, 1:1,000, and 1:2,000 dilutions), toluquinol (33.3, 100, and 300 μg/ml), gentisyl alcohol (33.3, 100, and 300 μg/ml), and patulin (33.3, 100, and 300 μg/ml) were applied to the rice by foliar spraying until runoff onto the leaves. Streptomycin sulfate (33.3, 100, and 300 μg/ml) and Tween 20 (500 μg/ml) were used as positive and non-treated controls, respectively.

To infect rice, scissors were dipped in the Xoo suspension and used to cut the tips (3–4 cm from the tips) of rice leaves. The rice plants were grown under a light regime of 12-h light/12-h dark at 28–36°C and 90% relative humidity. Disease severity (DS) was recorded 14 days after bacterial inoculation (DAI). The experiment included three biological replicates (three leaves per treatment), and the entire experiment was performed twice.

### 2.9. Disease control efficacy against bacterial wilt of tomato

Disease control efficacy against tomato bacterial wilt was examined as described in a previous study (Im et al., 2020). Tomato seeds (Seokwang, Farm Hannong Co, Seoul, Republic of Korea) were grown in pots (4.5 cm in diameter) containing horticulture nursery soil (Punong Co., Gyeongju, Republic of Korea) until the tomato plants reached the 4–5 true-leaf stage. The plants were then transplanted into pots (7 cm in diameter and 6 cm in height) and grown until the 7–8 true-leaf stage.

*Ralstonia solanacearum*, the causative agent of bacterial wilt, was cultured in TSA at 30°C for 2 days, and the cells were harvested using sterile water and a cell scraper. The cell density was measured using a UV spectrophotometer at 600 nm, and the bacterial inoculum suspension was adjusted to 1.5 × 10^8^ CFU/ml. One day before the inoculation of Rs, 20 ml of diluted T-WP (1:1,000, 1:2,000, and 1:4,000 dilutions) and T-SL (1:500, 1:1,000, and 1:2,000 dilutions) were applied to the tomato plants by soil drenching. The commercial pesticide Sungbocycline (oxytetracycline calcium alkyltrimethyl ammonium 17%, 1:1,000 dilution in accordance with manufacturer's recommendations; Sungbo Chemicals Co, LTD., Seoul, Republic of Korea) and Tween 20 (250 μg/ml) were used as positive and non-treated controls, respectively.

Twenty milliliters of the Rs suspension were drenched into the soil, and the plants were maintained at 30°C and 95% humidity for 2 weeks. DS was recorded at 9 and 14 DAI. The experiment included three biological replicates (three plants per treatment), and the entire experiment was performed twice.

### 2.10. Evaluation of the disease control efficacy

To evaluate the disease control efficacy against rice leaf blight and tomato bacterial wilt, DS was visually estimated on a 6-point scale (0 = no symptoms, 1 = one leaf partially wilted, 2 = one to two leaves wilted, 3 = two of three leaves wilted, 4 = four or more leaves wilted, and 5 = death of the entire plant) as described in a previous study (Winstead and Kelman, 1952). The control efficacy values for the individual plant diseases were calculated as follows:


$$\begin{array}{l} \%\;Control\;value\;=\\ \;100\;\times \;\left[\left(DS\;of\;control\;-DS\;of\;treatment\right)\;/\;DS\;of\;control\right] \end{array}$$

### 2.11. Statistical analysis

Experimental data were analyzed using a one-way or two-way analysis of variance, followed by Tukey's multiple comparison tests or the Bonferroni post-test (GraphPad Prism 5.0).

## 3. Results

### 3.1. Screening marine fungi for antibacterial activity *in vitro* and *in vivo*

The *in vitro* antibacterial activities of the marine fungi were studied against At, Rs, and Xap. Among the tested fungal strains, UL-Ce9 isolated from the marine alga, *Ceramium* sp., exhibited the highest antibacterial activity against At, Rs, and Xap. The MIC value of UL-Ce9 culture broth was 5% (v/v) against all the phytopathogenic bacteria examined.

The culture broth of fungi showing high antibacterial activities *in vitro* was subjected to an *in vivo* antibacterial activity test against the bacterial wilt of tomato. The uninoculated tomato plants did not show any wilting symptoms. In contrast, the tomato plants inoculated with Rs displayed typical wilting symptoms. This indicates that the tomato plants were infected with Rs. Among the marine fungi examined, the culture broth (1:5 dilution) of UL-Ce9 completely suppressed the disease (100% ± 0.00 disease control efficacy). Moreover, the disease control efficacies of UL-Ce9 filtrates at dilutions of 1:15 and 1:45 were 83.3 ± 2.3% and 76.2 ± 3.3%, respectively (Supplementary Figure 1).

### 3.2. Identification of the marine fungal strain UL-Ce9

**Figure 1:**
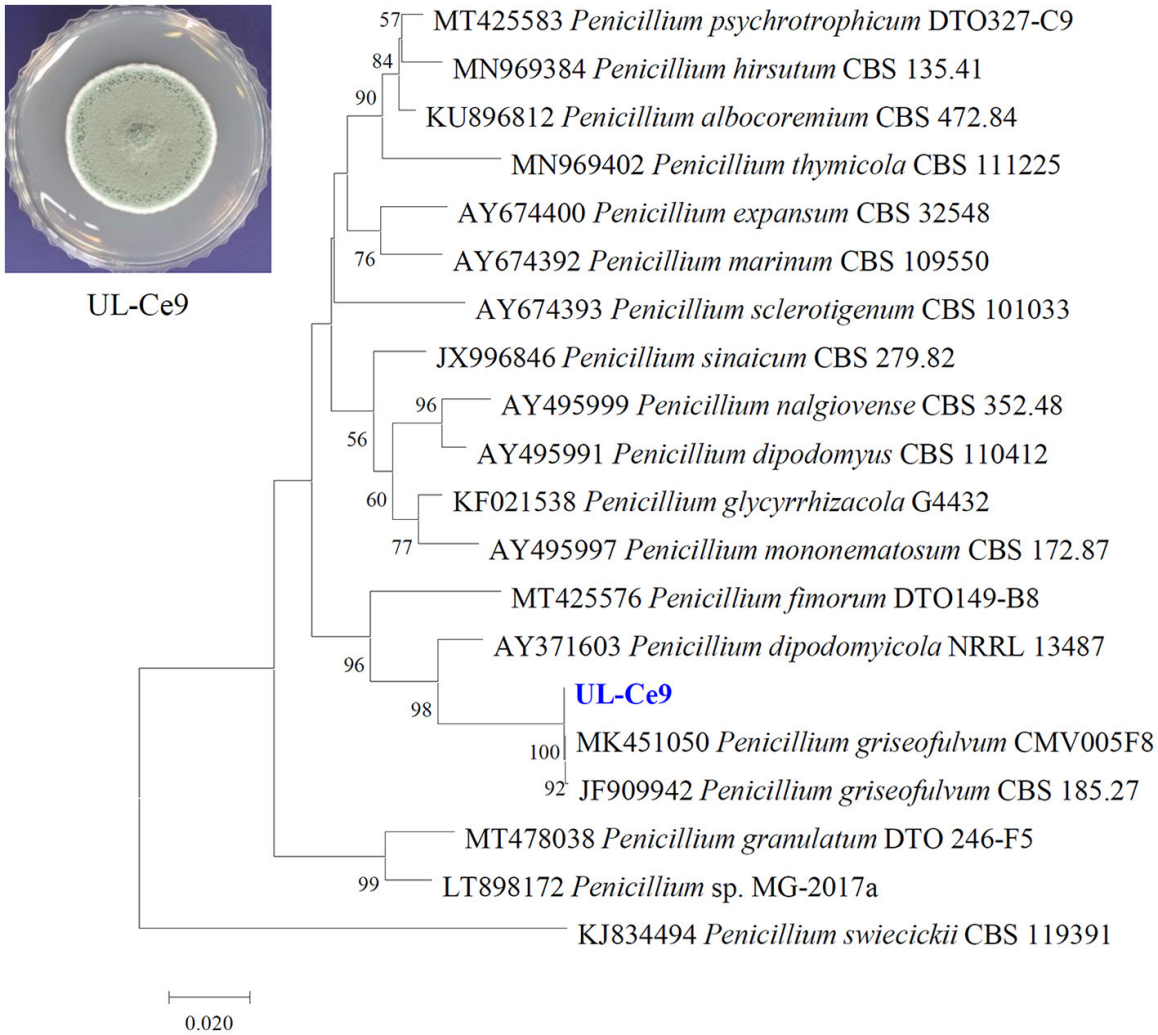
Identification of a marine fungal strain UL-Ce9. The plate picture shows a colony of UL-Ce9 grown on PDA at 25°C for 7 days. A neighbor-joining phylogenetic tree was generated using β-tubulin sequences of UL-Ce9 and closely related fungal species based on sequence similarity. Numbers at nodes indicate percent bootstrap values from 1,000 replications (values < 50% are not shown). The scale bar indicates the number of nucleotide substitutions per site.

UL-Ce9 was isolated from the blades of the marine alga *Ceramium* species. The UL-Ce9 β-tubulin gene sequence (deposited in GenBank; accession number OQ559567) showed the highest nucleotide similarity with that of *Penicillium griseofulvum* CMV005F8 (99.75%) and the *P. griseofulvum* type strain CBS 185.27 (99.24%; *E*-values = 0). The UL-Ce9 β-tubulin sequence was also closely related to *Penicillium dipodomyicola* NRRL 13487 with 94.64% sequence similarity (*E*-value = 1e-167). A neighbor-joining phylogenetic analysis using the β-tubulin sequence showed that UL-Ce9 formed a clade with these two *P. griseofulvum* strains, supported by a 100% bootstrap value. Therefore, UL-Ce9 was identified as *P. griseofulvum* (Figure 1).

### 3.3. Characterization of antibacterial metabolites from *P. griseofulvum* strain UL-Ce9

**Figure 2:**
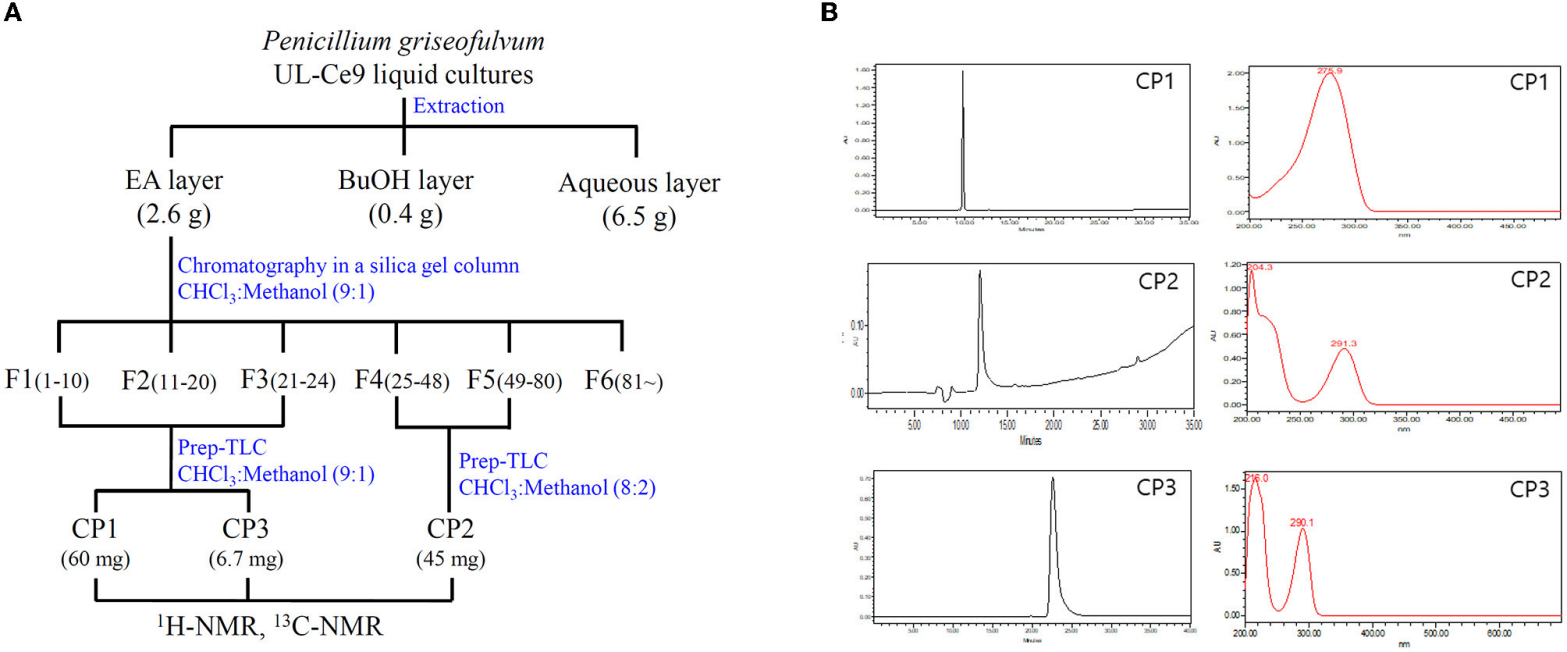
Isolation and purification of antibacterial metabolites from UL-Ce9. **(A)** The procedure to identify antibacterial metabolites of UL-Ce9. For the extraction of antibacterial compounds from UL-Ce9, ethyl acetate (EA), butanol (BuOH), and water (aqueous) were used. The EA extract was purified by a series of chromatography, and the resulting fractions were tested for their antibacterial activities. A total of six fractions were subjected to prep-TLC, and the obtained three compounds (C1–C3) were identified using NMR analysis. **(B)** The purities of C1–C3 were validated by HPLC at 254 nm.

The antibacterial activities of the EA, butanol, and water extracts of UL-Ce9 were examined against Rs *in vitro*, and the MIC values were 3.9, 7.8, and 250 μg/ml, respectively. As the EA extract showed the highest antibacterial activity, it was used for the isolation, purification, and identification of the antibacterial compounds of *P. griseofulvum* (Figure 2A).

More than 81 fractions obtained *via* chromatography were grouped into six fractions (F1–F6) based on separation patterns from the TLC analysis (Figure 2A). The antibacterial activities of F1 (210 mg), F2 (186 mg), F3 (250 mg), F4 (120 mg), F5 (162 mg), and F6 (250 mg) were assessed against Rs, and the MIC values were 31.2, 3.9, 3.9, 3.9, and 3.9 μg/ml for F1, F2, F3, F4, and F5, respectively. F6 did not show any antibacterial activity against Rs.

Compounds **1** (CP**1**) and **2** (CP**2**) were purified from fractions F13 and F45 by prep-TLC. Active CP**1** and CP**2** displayed one major spot in the TLC analysis, which was detected at an *R*_*f*_ value of 0.7 and 0.5, respectively. The purities of CP**1** and CP**2** were validated by HPLC at 254 nm, and the absorption maxima of CP**1** and **2** were observed at 276 and 291 nm, respectively (Figure 2B).

**Figure 3:**
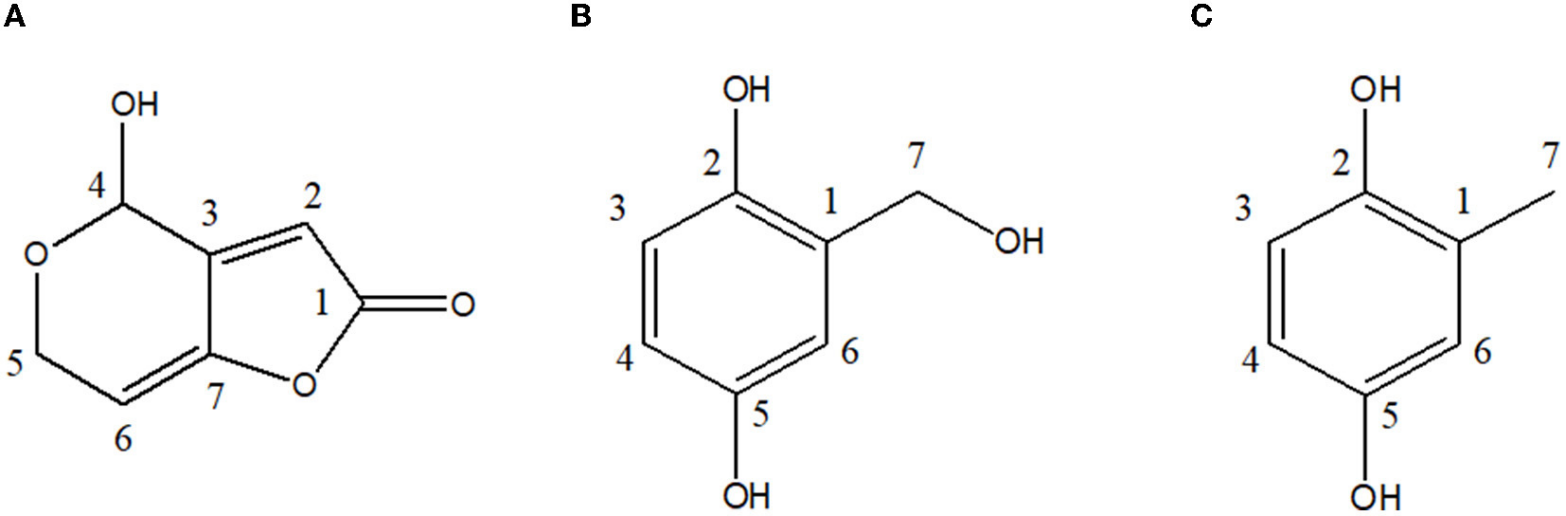
Chemical structures of antibacterial metabolites identified from UL-Ce9. **(A)** Patulin, **(B)** gentisyl alcohol, and **(C)** toluquinol.

The liquid chromatography-electrospray ionization-mass spectrometry (LC-ESI-MS) of CP**1** displayed an ion peak [M + H]^+^ at *m/z* 155.1, suggesting a molecular formula of C_7_H_5_O_4_, similar to that of patulin. Further evidence was obtained from the two proton signals at δ 4.53 (1H, dd, 16.8 and 3.0 Hz, and H-5a) and δ 4.34 (1H, dd, 16.8 and 3.0 Hz, and H-5b) in the ^1^H-NMR spectrum and the signals of one oxygenated methine at δ 87.94 (C-4) in the ^13^C-NMR spectrum (Table 2). Two methine protons at δ 6.21 (1H, m, and H-2) and δ 6.19 (1H, m, and H-6) were attached to a double bond in the ^1^H-NMR spectrum, and one carbon showed resonance at δ 168.78 (C-1) in the ^13^C-NMR spectrum. In the ^1^H-NMR spectrum, the singlet signal was observed at δ 5.92 (1H and s; Figure 3). These data suggest that CP**1** is patulin (Yoo and Park, 1995).

**Table 2:** NMR data for the antibacterial metabolites of *P. griseofulvum* strain UL-Ce9.

**Compound**	**Carbon**	**^1^H**	**^13^C**
Patulin	1	–	168.78
	2	6.21 (m)	109.55
	3	–	145.6
	4	5.92 (s)	87.94
	5a	4.53 (dd, 16.8: 3.0)	58.63
	5b	4.34 (dd, 16.8: 3.0)	58.63
	6	6.19 (m)	109.33
	7	–	152.33
Gentisyl alcohol	1	–	129.7
	2	–	149.1
	3	6.61 (d, 8.55)	116.8
	4	6.54 (dd, 3.0: 8.55)	115.6
	5	–	151.3
	6	6.75 (d, 2.75)	115.9
	7	4.59 (CH2, s)	61.2
	7-OH	4.96 (s)	–
Toluquinol	1	–	125.7
	2	–	147.0
	3	6.84 (dd, 8.60, 2.88)	117.2
	4	6.59 (dd, 8.60, 0.46)	114.3
	5	–	149.5
	6	6.53 (dd, 2.88, 0.46)	118.9
	7	2.15 (s)	15.4

The ESI-MS of CP**2** displayed an ion peak [M]^+^ at *m/z* 140, suggesting a molecular formula of C_7_H_8_O_3_, similar to that of gentisyl alcohol. The ^1^H-NMR showed aromatic protons at δ 6.61 (1H, d, 8.55, and H-3), 6.54 (1H, dd, 3.0, 8.55, and H-4), and 6.75 (d, 2.75, H-6) and methylene protons at δ 4.59 (2H, s, and H-7; Table 2). These data displayed phenolic hydroxyl groups at δ 4.96 (s and 7-OH). The presence of an OH group at C-2 and C-5 was confirmed by the fragment ion at *m/z* 122 (100; Figure 3). Based on these data, CP**2** was identified as gentisyl alcohol (Ishikawa et al., 1986).

Compound **3** showed one spot in the TLC analysis with an *R*_*f*_ value of 0.6 when irradiated with UV light at 254 and 365 nm. CP3 showed the maximum UV absorption at 290 nm (Figure 2B) by HPLC analysis. The ^1^H-NMR showed aromatic protons at δ 6.84 (1H, dd, 8.60, 2.88, and H-3), 6.59 (1H, dd, 8.6, 0.46, and H-4), and 6.53 (dd, 2.88, 0.4, and H-6) and a methyl group at δ 2.15 (3H, s, and H-7) (Table 2 and Figure 3). Based on these data, CP**3** was identified as toluquinol (Huang et al., 1989).

### 3.4. *In vitro* antibacterial activity of patulin, gentisyl alcohol, and toluquinol

Because patulin, gentisyl alcohol, and toluquinol are associated with patulin biosynthesis (Puel et al., 2010), we investigated the antibacterial activities of related metabolites (*m*-cresol, *m*-hydroxybenzyl alcohol, *m*-hydroxybenzaldehyde, *m*-hydroxybenzoic acid, gentisyl aldehyde, and gentisic acid) *in vitro* (Table 3).

**Table 3:** MIC values of patulin, gentisyl alcohol, toluquinol, and other metabolites of the patulin biosynthetic pathway.

**Phytopathogenic bacteria (abbreviations)**	**Minimum inhibitory concentration (MIC**, μg/ml)^*^									
	**Patulin**	**Gentisyl alcohol**	**Toluquinol**	***m*** **-cresol**	***m*** **-hydroxy benzyl alcohol**	***m*** **-hydroxy benzaldehyde**	***m*** **-hydroxy benzoic acid**	**Gentisyl aldehyde**	**Gentisic acid**	**Streptomycin sulfate**
Aac	25.0	12.5	12.5	>200	>200	>200	>200	200	>200	>200
Ak	50.0	25.0	6.25	>200	>200	>200	>200	100	>200	6.25
At	25.0	>200	50.0	>200	>200	>200	>200	100	>200	100
Bg	12.5	100	100	>200	>200	>200	>200	100	>200	25.0
Cmm	3.13	25.0	100	>200	>200	>200	>200	100	>200	12.5
Ea	12.5	25.0	25.0	>200	>200	>200	>200	100	>200	1.56
Ep	12.5	200	200	>200	>200	>200	>200	>200	>200	1.56
Pcc	12.5	>200	50.0	>200	>200	>200	>200	50.0	>200	12.5
Pc	25.0	>200	100	>200	>200	>200	>200	50.0	>200	>200
Psa	100	>200	200	>200	>200	>200	>200	200	>200	6.25
Psl	50.0	>200	200	>200	>200	>200	>200	200	>200	6.25
Rs	3.13	6.25	12.5	200	>200	>200	>200	12.5	>200	6.25
Xap	6.25	6.25	3.13	200	>200	>200	>200	50.0	>200	12.5
Xac	12.5	50.0	12.5	>200	>200	>200	>200	50.0	>200	12.5
Xe	12.5	50.0	6.25	>200	>200	>200	>200	25	>200	12.5
Xoo	3.13	3.13	0.78	200	>200	100	>200	12.5	12.5	3.13

Aac, *Acidovorax avenae* subsp. *cattleyae*; Ak, *Acidovorax konjaci*; At, *Agrobacterium tumefaciens*; Bg, *Burkholderia glumae*; Cmm, *Clavibacter michiganensis* subsp. *michiganensis*; Ea, *Erwinia pyrifoliae*; Pcc, *Pectobacterium carotovora* subsp. *carotovora*; Pc, *Pectobacterium chrysanthemi*; Psa, *Pseudomonas syringae* pv. *actinidiae*; Psl, *Pseudomonas syringae* pv. *lachrymans*; Rs, *Ralstonia solanacearum*; Xap, *Xanthomonas arboricola* pv. *pruni*; Xac, *Xanthomonas axonopodis* pv. *citri*; Xe, *Xanthomonas euvesicatoria*; Xoo, *Xanthomonas oryzae* pv. *oryzae*.

^*^The results from three biological replicates were identical (standard deviation = 0).

Overall, compared with patulin, gentisyl alcohol, and toluquinol, the other six metabolites exhibited lower antibacterial activities against all phytopathogenic bacteria examined. In particular, the MIC values of *m*-cresol, *m*-hydroxybenzyl alcohol, *m*-hydroxybenzaldehye, *m*-hydroxybenzaldehyde, and gentisic acid were over 200 μg/ml, except 100 μg/ml for *m*-hydroxybenzaldehyde and 12.5 μg/ml for gentisic acid against Xoo. Gentisyl aldehyde showed higher antibacterial activity than those of the other five metabolites, with MIC values similar to those of toluquinol against *B. glumae* (Bg), *Pectobacterium carotovora* subsp. *carotovora* (Pcc), *P. chrysanthemi* (Pc), *P. syringae* pv. *actinidiae* (Psa), and Rs.

Patulin showed the highest antibacterial activities against most phytopathogenic bacteria except *A. avenae* subsp. *cattleyae* (Aac), *A. konjaci* (Ak), Xap, *X*. *euvesicatoria* (Xe), and Xoo. The lowest MIC values (3.13 μg/ml) of patulin were observed against *C*. *michiganensis* subsp. *michiganensis* (Cmm), Rs, and Xoo, followed by Xap (MIC 6.25 μg/ml). The highest antibacterial activity of gentisyl alcohol was observed against Xoo (MIC 3.13 μg/ml). In addition, it showed strong inhibitory effects on the growth of Rs and Xap (MIC 6.25 μg/ml). A metabolite with the highest antibacterial activity against Xoo was toluquinol (0.78 μg/ml). Compared with gentisyl alcohol, toluquinol showed higher activity against nine phytopathogenic bacteria (Ak, At, Pcc, Pc, Psa, Psl, Xap, Xe, and Xoo).

Due to the potentially negative effects of patulin on human health (Pal et al., 2017), we prepared two types of pesticide formulations, namely, T-SL and T-WP, using toluquinol to assess the disease control efficacy against bacterial plant diseases.

### 3.5. Suppression of bacterial leaf blight of rice by toluquinol

**Figure 4:**
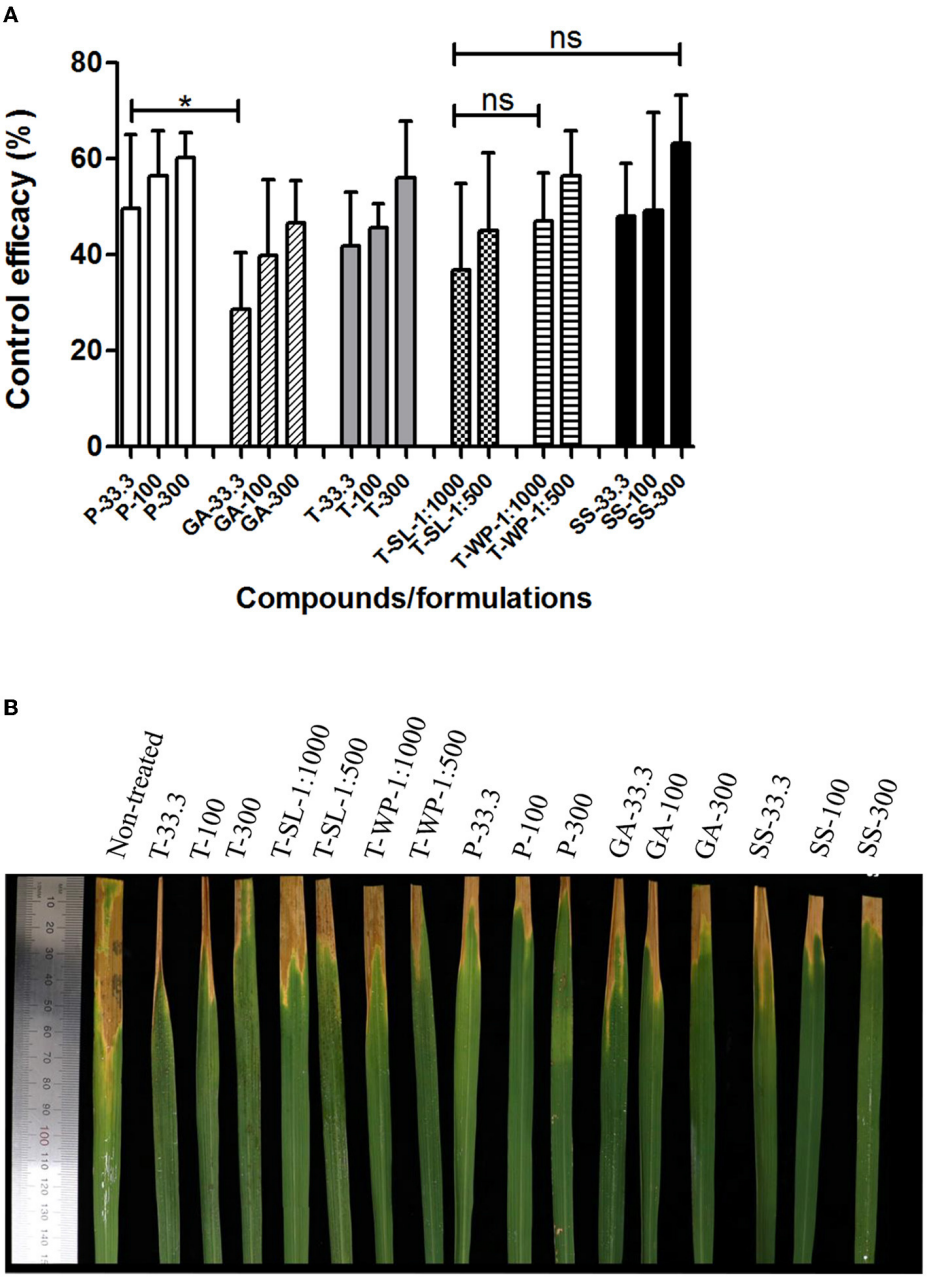
Suppression of bacterial leaf blight of rice by toluquinol. Control efficacies of patulin (P), gentisyl alcohol (GA), and toluquinol (T) at 33.3, 100, and 300 μg/ml, and two formulations of toluquinol, soluble concentrate (T-SL) and wettable powder (T-WP), at 1:1,000 and 1:500 dilutions were investigated against leaf blight of rice relative to the control, streptomycin sulfate (SS). **(A)** The control efficacies of tested compounds/formulations are presented as the mean ± standard deviation from five replicates. **(B)** Rice leaves were treated with each compound/formulation 1 day before inoculation with Xoo. Disease severity was evaluated in comparison with that of the untreated control to calculate control efficacy. “*” and “ns” indicate statistically significant (*P* < 0.05) and non-significant (*P* > 0.05), respectively.

Patulin, gentisyl alcohol, and toluquinol, as well as T-SL and T-WP, were evaluated for their efficacy against rice leaf blight caused by Xoo (Figure 4). Overall, all metabolites and formulations effectively suppressed the development of leaf blight of rice, similar to the positive control, streptomycin sulfate, except for gentisyl alcohol at 33.3 μg/ml. Gentisyl alcohol was less effective than patulin to suppress leaf blight of rice at 33.3 μg/ml (*P* < 0.05). There were no differences in the efficacies of different concentrations of each compound/formulation. For example, the efficacies of T-SL were 36.9 ± 17.9% and 45.0 ± 16.2% at 1:1,000 and 1:500 dilutions, respectively, which were not statistically different (*P* > 0.05). Similarly, T-WP exhibited control efficacies of 47.1 ± 10.0% and 56.4 ± 9.4% at 1:1,000 and 1:500 dilutions. At 100 μg/ml, the control efficacies of patulin, gentisyl alcohol, and toluquinol were 56.4 ± 9.4%, 39.8 ± 15.8%, and 45.6 ± 5.0%, respectively. Notably, patulin showed phytotoxicity in rice plants at 300 μg/ml, causing leaf desiccation and leaf spots on the leaves 2 days after treatment. These data suggest that toluquinol and its formulations exert strong suppressive effects on leaf blight of rice, similar to the currently used chemical (streptomycin) for disease management.

### 3.6. Suppression of bacterial wilt of tomato by toluquinol

**Figure 5:**
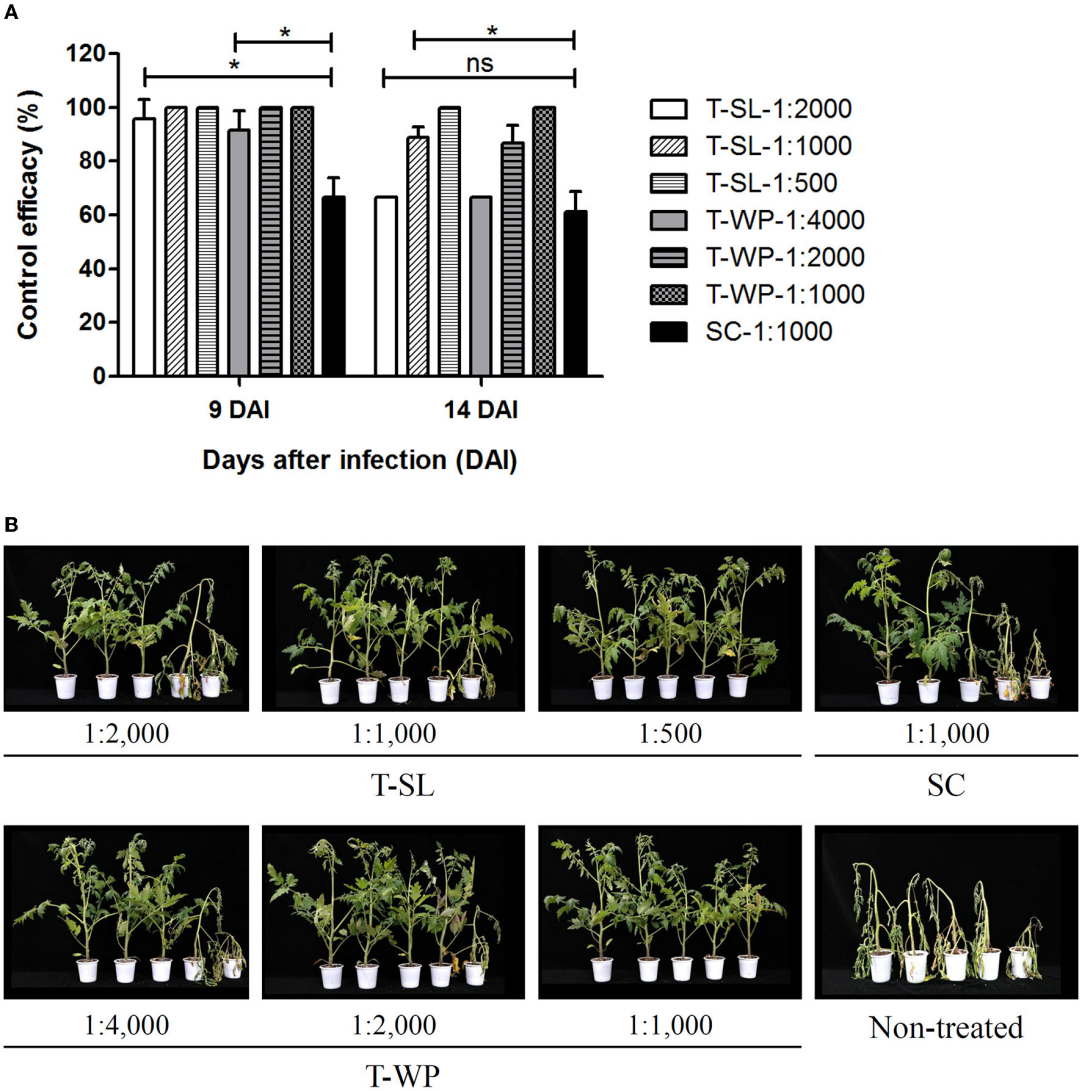
Suppression of bacterial wilt of tomato by toluquinol. The control efficacies of two formulations of toluquinol, soluble concentrate (T-SL) and wettable powder (T-WP), were investigated against bacterial wilt of tomato relative to the control, a commercial pesticide, Sungbocycline (SC). **(A)** The control efficacies of the tested formulations are presented as the mean ± standard deviation from three replicates. **(B)** Tomato plants were treated with each formulation at different concentrations 1 day before inoculation with *Ralstonia solanacearum* (plant images taken at 14 DAI). Disease severity was evaluated in comparison with that of the untreated control to calculate control efficacy. “*” and “ns” indicate statistically significant (*P* < 0.05) and non-significant (*P* > 0.05), respectively.

The disease control efficacy of toluquinol against tomato bacterial wilt caused by Rs was investigated using two toluquinol formulations, T-SL and T-WP (Figure 5). Overall, both the toluquinol formulations showed higher control efficacies, even at the lowest concentrations, compared with that shown by the positive control, a commercial pesticide Sungbocycline (oxytetracycline), at 9 DAI (*P* < 0.05). At 14 DAI, the control efficacies of the most diluted T-SL (1:2,000) and T-WP (1:4,000) formulations slightly decreased compared with those at 9 DAI, similar to that of Sungbocycline (*P* > 0.05). In contrast, at higher concentrations, T-SL (1:500) and T-WP (1:1,000) completely suppressed the bacterial wilt of tomato (100 ± 0.00% efficacy) at both 9 DAI and 14 DAI, whereas the control efficacy of Sungbocycline was 66.7 ± 7.2% and 51.11 ± 7.7%, respectively. These results suggest that toluquinol suppresses bacterial wilt of tomato more effectively than the currently used pesticides.

## 4. Discussion

This study aimed to identify antibacterial compounds produced by marine fungi that could be used for the development of effective and novel pesticides. We identified three antibacterial fungal metabolites, namely, patulin, gentisyl alcohol, and toluquinol, in the marine alga-derived *P. griseofulvum* strain. These compounds are associated with the biosynthesis of patulin, which is a well-known mycotoxin produced by several species of *Penicillium, Aspergillus, Paecilomyces*, and *Byssochlamys* (Puel et al., 2010). It has been reported that one of the potential reasons for mycotoxin production in fungi is to suppress phytopathogenic bacteria that share host plants (Venkatesh and Keller, 2019). For example, the causal agent of the bakanae disease in rice seedlings, *Fusarium fujikuroi*, produces antibacterial mycotoxins such as bikaverin and beauvericin against *R. solanacearum* (Spraker et al., 2018).

Among patulin, gentisyl alcohol, and toluquinol, we observed the highest antibacterial activity against the 16 phytopathogenic bacteria in patulin (Table 3). However, patulin is considered to negatively affect human health (Puel et al., 2010), and toluquinol showed higher antibacterial activity than gentisyl alcohol. Therefore, we hypothesized that toluquinol might possess greater potential as a candidate for pesticide development than either patulin or gentisyl alcohol.

Toluquinol is also known as methylhydroquinone, 2,5-toluenediol, or 2,5-dihydroxytoluene and is commercially available. Toluquinol has been studied in *P. griseofulvum* (Sekiguchi and Gaucher, 1977), *Hydropisphaera erubescens* (Carey and Nari, 1979), *Aspergillus clavatus* (current name: *Neosartorya clavata*; Artigot et al., 2009), *Penicillium* sp. (Garcia-Caballero et al., 2013), and *Aspergillus* sp. (Kim et al., 2015). Toluquinol isolated from two marine fungal strains has been reported to suppress angiogenesis. For example, toluquinol from the marine *Penicillium* sp. HL-85-ALS5-R004 strain inhibits the growth of bovine endothelial and human tumor cells (Garcia-Caballero et al., 2013). Moreover, toluquinol from the marine algae-derived *Aspergillus* sp. MFA292 strain also inhibits angiogenesis in human endothelial cells (Kim et al., 2015). To the best of our knowledge, this is the first report of the antibacterial activity of toluquinol against phytopathogenic bacteria.

Other six metabolites (*m*-cresol, *m*-hydroxybenzyl alcohol, *m*-hydroxybenzaldehyde, *m*-hydroxybenzoic acid, gentisyl aldehyde, and gentisic acid) in the patulin biosynthetic pathway (Artigot et al., 2009) showed much lower antibacterial activities than patulin, gentisyl alcohol, and toluquinol (Table 3). In the first step of patulin synthesis, 6-methylsalicylic acid (6MSA) is produced by the condensation of acetyl-CoA and malonyl-CoA (Puel et al., 2010). 6MSA is converted to *m*-cresol by 6MSA decarboxylase, followed by the transformation of *m*-cresol into *m*-hydroxybenzyl alcohol or toluquinol by two distinct cytochrome P450 enzymes, namely, CYP619C3 and CYP619C2 (Artigot et al., 2009). The enzyme CYP619C2, which is involved in the formation of toluquinol from *m*-cresol, is also responsible for the hydroxylation of *m*-hydroxybenzyl alcohol to yield gentisyl alcohol. Unlike gentisyl alcohol, toluquinol is not metabolized to patulin, suggesting that it is a co-metabolite and not an intermediate of patulin (Puel et al., 2010).

Toluquinol formulations were prepared as T-SL and T-WP. T-SL is an aqueous solution and more environmentally friendly than other formulations (Chou et al., 2022). T-WP is a solid formulation suspended in water. T-SL and T-WP are pesticide formulations commonly used to control bacterial plant diseases. Based on our data, we did not observe any differences in the suppressive effects of T-SL and T-WP on the two plant diseases (Figures 4, 5). This suggests that toluquinol can be formulated as either a T-SL or T-WP with similar control efficacy levels.

In the present study, two representative bacterial plant diseases were selected to evaluate the disease control efficacy of toluquinol: leaf blight of rice and bacterial wilt of tomato. Singh et al. (1980) reported that copper compounds and antibiotics, including streptomycin, are not effective in controlling leaf blight of rice. As toluquinol formulations suppressed these significant bacterial diseases similarly or more effectively than the existing chemicals (streptomycin and oxytetracycline; Figures 4, 5), toluquinol could be a candidate for the development of new pesticides targeting bacterial plant diseases. Future studies should investigate the efficacy of toluquinol under various field conditions using multiple cultivars and against additional bacterial plant diseases.

## 5. Conclusion

In this study, three antibacterial metabolites (patulin, gentisyl alcohol, and toluquinol) against a variety of phytopathogenic bacteria were identified from a marine *P. griseofulvum* strain. Gentisyl alcohol and toluquinol are an intermediate and a co-metabolite of the mycotoxin patulin, respectively. Toluquinol showed higher antibacterial activity against phytopathogenic bacteria *in vitro* than gentisyl alcohol and suppressed bacterial wilt of tomato and leaf blight of rice similarly or more effectively compared with the currently used pesticides. Given that only limited approaches to control bacterial plant diseases are available, toluquinol could be used to develop novel and effective pesticides for crop protection.

## Data availability statement

The datasets presented in this study can be found in online repositories. The names of the repository/repositories and accession number(s) can be found at: https://www.ncbi.nlm.nih.gov/nuccore/OQ559567.

## Author contributions

DC and HN performed experiments and wrote the manuscript. NY designed the experiments and analyzed the data. W-JY, YK, and SB assisted with the collection of marine samples and fungal identification. GC and J-CK conceived the original study and supervised the project. All authors have read and approved the submitted version of the manuscript.
