# Supplementary figures and images for: In vitro and in vivo antimicrobial activity of the fungal metabolite toluquinol against phytopathogenic bacteria
Source: Front Microbiol. 2023 Jul 31;14:1221865. doi: 10.3389/fmicb.2023.1221865 (PMC10424571; doi:10.3389/fmicb.2023.1221865)

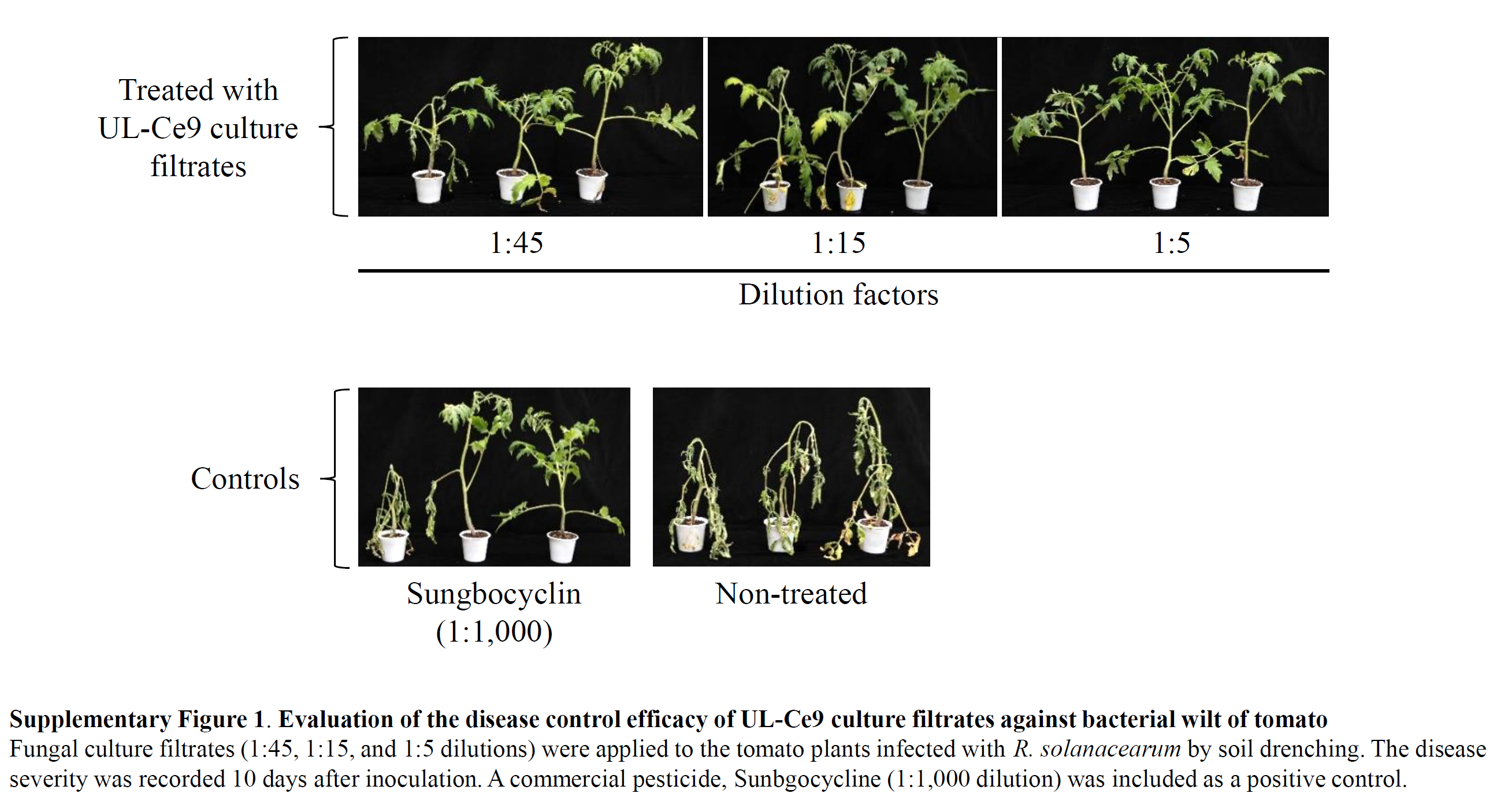

Supplement: Supplementary file 1 [file Image_1.TIF]
